# A Bureau of Autopsy

**Published:** 1894-09-15

**Authors:** 


### A BUREAU OF AUTOPSY.

In the year 1881 there was founded at Milan a unique and curious institution, which has been at work ever since. This was the Loria Bureau of Autopsy. It was founded, and placed under the direction of Professor A. Verga, for the use of the inhabitants of Milan. That is to say, either courts of law or the family of a deceased person can have a complete examination and report in cases where a death has occurred under suspicious or strange circumstances. All autopsies are recorded, and may be consulted for purposes of study. The Bureau is intended to serve two purposes—the advancement of knowledge, and to remove one objection to cremation raised on the grounds that crime may be less easy of detection when the deceased is effectually placed beyond the reach of examination.

